# Relationship between temperature variability and daily hospitalisations in Hong Kong over two decades

**DOI:** 10.7189/jogh.13.04122

**Published:** 2023-10-13

**Authors:** Kehang Li, Yawen Wang, Xiaoting Jiang, Conglu Li, Jinjian Chen, Yiqian Zeng, Shi Zhao, Janice Ying-en Ho, Jinjun Ran, Lefei Han, Yuchen Wei, Eng Kiong Yeoh, Ka Chun Chong

**Affiliations:** 1 Jockey Club School of Public Health and Primary Care, The Chinese University of Hong Kong, Hong Kong Special Administrative Region, China; 2 Centre for Health Systems and Policy Research, The Chinese University of Hong Kong, Hong Kong Special Administrative Region, China; 3 Clinical Trials and Biostatistics Laboratory, Shenzhen Research Institute, The Chinese University of Hong Kong, Shenzhen, China; 4 Division of Landscape Architecture, Department of Architecture, Faculty of Architecture, The University of Hong Kong, Hong Kong, Hong Kong Special Administrative Region, China; 5 School of Public Health, Shanghai Jiao Tong University School of Medicine, Shanghai, China; 6 School of Global Health, Chinese Center for Tropical Diseases Research, Shanghai Jiao Tong University School of Medicine, Shanghai, China

## Abstract

**Background:**

Studies have highlighted the impacts of temperature variability (TV) on mortality from respiratory diseases and cardiovascular diseases, with inconsistent results specifically in subtropical urban areas than temperate ones. We aimed to fully determine TV-associated health risks over a spectrum of diseases and various subgroups in a subtropical setting.

**Methods:**

Using inpatient data from all public hospitals in Hong Kong from 1999 to 2019, we examined the TV-hospitalisation associations by causes, ages, and seasons by fitting a quasi-Poisson regression. We presented the results as estimated percentage changes of hospitalisations per interquartile range (IQR) of TV.

**Results:**

TVs in exposure days from 0-5 days (TV_0-5_) to 0-7 days (TV_0-7_) had detrimental effects on hospitalisation risks in Hong Kong. The overall population was significantly affected over TV_0-5_ to TV_0-7_ in endocrine, nutritional and metabolic (from 0.53% to 0.58%), respiratory system (from 0.38% to 0.53%), and circulatory systems diseases (from 0.47% to 0.56%). While we found no association with seasonal disparities, we did observe notable disparities by age, highlighting older adults’ vulnerability to TVs. For example, people aged ≥65 years experienced the highest change of 0.88% (95% CI = 0.34%, 1.41%) in hospitalizations for injury and poisoning per IQR increase in *TV_0-4_*.

**Conclusions:**

Our population-based study highlighted that TV-related health burden, usually regarded as minimal compared to other environmental factors, should receive more attention and be addressed in future relevant health policies, especially for vulnerable populations during the cold seasons.

The impact of ambient temperature on health outcomes has been well-recognised, with many studies showing that extreme cold and heat are associated with increased mortality and morbidity across numerous diseases, particularly among vulnerable and elderly populations [1,2]. Besides ambient temperature, temperature variability (TV) is an important meteorological indicator that poses health risks to human and living systems due to their limited adaptation capacity, which refers to an individual's ability to acclimate and perform under varying external conditions while mitigating environmental effects. The human thermoregulatory system’s inadequate response to intense temperature changes occurring within a short period is a common issue [3]. It may cause a disturbance in the circulation and immune system, lifts in cardiovascular workload, and severe inflammatory reactions [4,5], leading to heat/cold stress-related illnesses and worsening health status. Furthermore, the ongoing climate variation caused by climate change is expected to exacerbate exposure to extreme TV, raising concerns about its associated health impacts.

Studies about the relationship between population health and TV primarily focus on short-term TVs, such as intra-day (diurnal temperature range) and inter-day (between 0-1 day) TV. Previous works demonstrated a significant mortality risk associated with short-term TV in many countries, particularly in hot regions [6,7]. However, evidence of TV-associated hospitalisation risks is less robust, with considerable gaps in the literature. Most studies examined the associations in hospitalisations for all-cause, cardiovascular diseases, and respiratory diseases [8-10]. For example, national studies in China and Brazil confirmed the adverse health effects of TVs at 0-1 day or intra-day TV on the previously mentioned diseases [9,11,12].

Although few studies explored the role of TV exposure in other common diseases [13,14], it is still unclear how long-term TV exposure, with a broader range of exposure days, is related to cause-specific hospitalisations from a variety of diseases. Moreover, the associations between TV and health outcomes are generally weaker than those observed for other environmental factors, such as ambient temperature and air pollution. Inconsistent patterns in prior findings suggest the notable geographical and socioeconomic heterogeneities in TVs' effects, especially in subtropical areas [4,6,15]. Compared to temperate areas, TVs in subtropical areas have large regional variations with relatively lower exposure levels [15], leading to more mixed and less significant estimated associations, necessitating further studies in subtropical areas. Hong Kong is a densely populated and developed setting with a subtropical climate, where temperatures increase and fluctuate due to climate change and urban heat island effects [16]. Here we examined the association between TV and cause-specific hospitalisation in Hong Kong from 1999 to 2019, stratifying the analyses by diseases, ages, and seasons to identify the vulnerable subpopulations to TV exposure and diseases with higher risks of TV-related burden in Hong Kong.

## METHODS

### Hospitalisation and meteorological data

We obtained inpatient data from the Hong Kong Hospital Authority that comprised patient-level information from all the public hospitals in Hong Kong, accounting for 83% of overall hospitalisations locally [17]. The data included age, sex, and principal diagnosis at discharge encoded by the International Classification of Diseases, Ninth Revision (ICD-9) [18]. We extracted hospital admissions of 14 categories of diseases from 1 January 1999 to 31 December 2019 and identified conditions by their ICD-9 codes (Table S1 in the **Online Supplementary Document**).

We retrieved data on daily air temperature of the same study period, including absolute daily maximum temperature (*T_max_*), daily mean temperature  (*T_mean_*), and absolute daily minimum temperature (*T_min_*) in °C from the open-access website of Hong Kong Observatory [19]. We then calculated TV exposures (from TV in 0-1 day to TV in 0-7 days) during the study period as the standard deviation of all the *T_min_* and *T_max_* during the consecutive exposure days; for example, the calculation of TV exposure for the current and following *N* days (*TV_0-N_*) is the standard deviation between *T_min-lag0_*, *T_max-lag0_*, *T_min-lag1_*, *T_max-lag1_*, …, *T_min-lagN_*, *T_max-lagN_* [12].

### Statistical analysis

We used a quasi-Poisson regression incorporating distributed lagged nonlinear model (DLNM) to examine the associations between 14 cause-specific hospitalisations and TV exposures from 0-1 day (*TV_0-1_*) to 0-7 days (*TV_0-7_*) in Hong Kong. DLNM is a modelling framework containing a detailed representation with additional time dimensions of exposure-response relationships, quantifying the delayed effects with high flexibility [20]. We assumed that TV has a linear exposure effect and daily temperatures have nonlinear and delayed effects on health. Moreover, we controlled for the confounding effects of seasonality, daily mean relative humidity, daily total rainfall, day of the week, and public holidays on hospitalisations in the model. We based the selection of variables, degrees of freedom and lags in the model on standard settings and previous studies [6,14,21], with the model formulation being as follows:

*Y_it_ ~ Poisson*(*μ_it_*)

*Log*(*μ_it_*)   *~ α* + *βTV_t_* + *ns*(*Time, 7 × year*) + *cb*(*T_mean_*) + *ns(RH,* 3) + *ns*(*RF,* 3) + *γDOW_t_* + *δHoliday_t_*

where *Y_it_* is the number of hospital admissions for disease *i* on day *t*, which follow the Poisson distribution with mean *μ_it_* and α is the intercept. Dependencies are described by components of TV in linear function with coefficient β and cross-basis matrix (*cb*( · )) of daily mean temperature *T_mean_* with 21 days of lag. We controlled the effect of relative humidity (*RH*) and rainfall (*RF*) using two natural cubic splines (denoted by *ns*( · )) with three degrees of freedom and adjusted the long-term trend and seasonality by the natural cubic spline function with seven degrees of freedom per year. Variables *DOW_t_* and *Holiday_t_* were the indicators for day of the week (1, 2, 3, …, 7) and public holidays (0,1), respectively, reducing the effects of short-term fluctuations.

We conducted subgroup analyses by stratifying the hospitalisations into five age groups (0-18 years, 19-49 years, 50-64 years, 65-79 years, ≥80 years) and three seasons by months (cold season: December to February, hot season: July to September, moderate season: March to May, October to November). We reported association measurements as percentage changes of hospitalisations per interquartile range (IQR) increase of TV exposures (TV_0-1_ to TV_0-7_) with 95% confidence intervals (CIs), using the calculation of relative risks (RR) per IQR increase in TV ((RR – 1) × 100%). RRs are determined as the exponential values of the coefficient β multiplied with the IQR of TV exposures (ie, *e^β^ ^IQR^*^(^*^TV^*^)^).

We conducted sensitivity analyses to examine the robustness of the estimates by switching the maximum lag days of *T_mean_* from 14 days to 28 days and degrees of freedom in smooth functions of *T_mean_* from 2 to 7. We also checked the impacts of cold spells and extreme heat on the association of interest. Cold spell and extreme heat are defined as periods over two continuous days with *T_mean_* lower than five percentiles/larger than 95 percentiles of daily mean temperatures over the study period in Hong Kong, respectively. We performed all statistical analyses using the *dlnm* and *stats* packages in R, version 4.0.2 (R Core Team, Auckland, New Zealand) [22,23].

## RESULTS

### Data description

There were 38 091 114 hospital admissions from 1 January 1999 to 31 December 2019. The most common diagnoses for admissions were genitourinary system diseases (13.81%), circulatory system diseases (12.96%), neoplasms (9.92%), and infectious and parasitic diseases (9.48%). About 54% of admissions were individuals over 65 years of age, and more admissions occurred in cold seasons than in hot and moderate seasons (Table S2 in the **Online Supplementary Document**).

The average daily mean temperatures during the study period (1999-2019) were 23.60°C for the whole year, 17.24°C for cold seasons, and 28.54°C for hot seasons. Average TVs ranged from 2.67°C to 2.84°C across TV_0-1_ to TV_0-7_ across the whole year, from 2.66°C to 3.02°C during cold seasons, and from 2.63°C to 2.72°C during hot seasons(**Table 1**).

**Table 1:** Summary of daily mean temperature and temperature variabilities in exposure days from 0-1 to 0-7 days by seasons in Hong Kong from 1999 to 2019*

	Mean (10^th^-90^th^ percentile)			
**Variable in °C**	**Overall**	**Cold seasons**	**Moderate seasons**	**Hot seasons**
*T_mean_*	23.60 (16.30-29.50)	17.24 (13.14-20.80)	23.41 (18.80-27.40)	28.54 (26.50-30.10)
TV_0-1_	2.67 (1.73-3.61)	2.66 (1.66-3.67)	2.67 (1.65-3.71)	2.72 (1.94-3.47)
TV_0-2_	2.67 (1.86-3.49)	2.70 (1.82-3.60)	2.68 (1.77-3.63)	2.65 (1.99-3.29)
TV_0-3_	2.71 (1.95-3.48)	2.78 (1.94-3.65)	2.73 (1.88-3.63)	2.64 (2.05-3.19)
TV_0-4_	2.75 (2.02-3.51)	2.85 (2.00-3.74)	2.78 (1.95-3.65)	2.63 (2.11-3.16)
TV_0-5_	2.78 (2.08-3.55)	2.91 (2.11-3.82)	2.82 (2.01-3.69)	2.64 (2.15-3.13)
TV_0-6_	2.81 (2.13-3.58)	2.97 (2.16-3.85)	2.85 (2.07-3.71)	2.65 (2.19-3.10)
TV_0-7_	2.84 (2.18-3.61)	3.02 (2.20-3.89)	2.89 (2.11-3.72)	2.65 (2.23-3.09)

TV_0-1_ – temperature variability at 0-1 day, TV_0-2_ – temperature variability at 0-2 days, TV_0-3_ – temperature variability at 0-3 days, TV_0-4_ – temperature variability at 0-4 days, TV_0-5_ – temperature variability at 0-5 days, TV_0-6_ – temperature variability at 0-6 days, TV_0-7_ – temperature variability at 0-7 days

*Cold seasons: December to February of next year. Moderate season: March to May, October to November. Hot seasons: July to September.

### Relationship between temperature variability and hospitalisations

**Figure 1:**
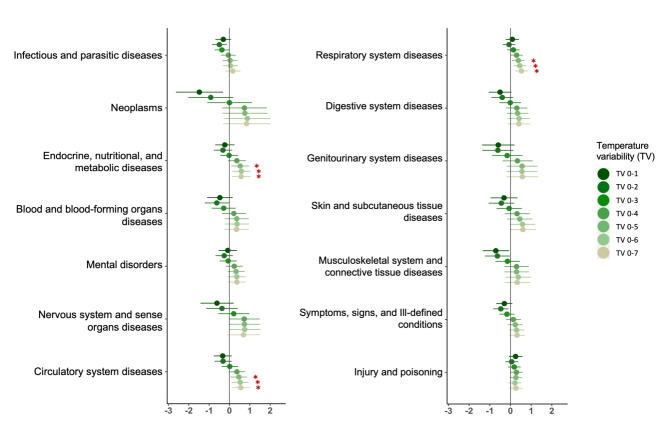
Percentage change (%) in cause-specific hospitalizations per interquartile increase in temperature variability (°C) in exposure days from 0-1 to 0-7 days in Hong Kong. Red stars highlighted the changes in hospitalisations significant at 95% confidence level.

Overall, we observed significant TV-associated hospitalisation risks in TV exposures from TV_0-4 _to TV_0-7_ and higher significant estimates in TV exposures at longer exposure days. All-cause hospitalisations were significantly associated with per IQR unit increase in TV, with an increase of 0.50% (95% CI = 0.02-0.98) in TV_0-6_ and 0.51% (95% CI = 0.02-1.00) in TV_0-7_ (Table S3 in the **Online Supplementary Document**). The TV effects on cause-specific hospitalisations varied between groups of diseases, but shared similar distributions of effect estimates across TV_0-1_ to TV_0-7_, where associations were stronger when exposure days of TV were longer (Figure 1). Specifically, we noticed significant hospitalisation changes of 0.53%-0.58% for endocrine, nutritional, and metabolic diseases, 0.47%-0.56% for circulatory system diseases, 0.38%-0.53% for respiratory system diseases, associated with per IQR unit increase in TV exposures from TV_0-5_ to TV_0-7_.

**Figure 2:**
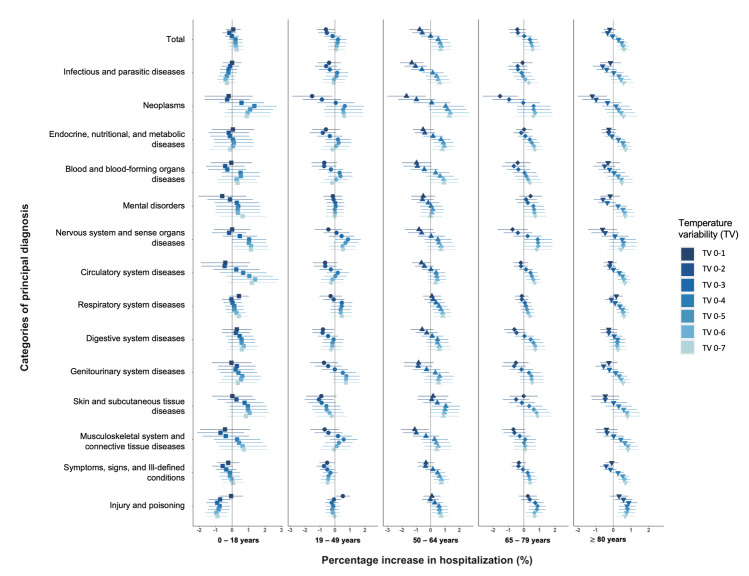
Percentage change (%) in all-cause and cause-specific hospitalisations per interquartile increase in temperature variability (°C) in exposure days from 0-1 to 0-7 days by age groups in Hong Kong. The points in different shapes draw the estimates with 95% CI of the associations among people aged 0-18 years (in shape square), 19-49 years (in shape circle), 50-64 years (in shape triangle), 65-79 years (in shape diamond), ≥80 years (in shape triangle down).

We found that the effects of TV on hospitalisation risks were age-dependent (**Figure 2**). In general, TV-associated risks were weaker among the younger age groups, meaning that only people aged ≥50 years experienced significant responses to TVs (TV_0-5_ to TV_0-7_) in all-cause hospitalisations (Table S3 in the **Online Supplementary Document**). The vulnerable groups to TVs varied between different cause-specific hospitalisations as well. Examine the highest estimates of cause-specific effects by age groups, people aged 50-64 years had greater susceptibility in skin and subcutaneous tissue diseases (1.06% on TV_0-4_; 95% CI = 0.08-2.05), endocrine, nutritional, and metabolic diseases (0.94% on TV_0-6_; 95% CI = 0.25-1.63), while those aged ≥65 years were found to be more vulnerable to injury and poisoning (0.88% on TV_0-4_; 95% CI = 0.34-1.41), musculoskeletal system and connective tissue diseases (0.84% on TV_0-7_; 95% CI = 0.24-1.44). Notably, we found TV effects on the nervous system and sense organs diseases to be strongest among people aged ≤18 years (1.15% on TV_0-7_; 95% CI = 0.07-2.24) and insignificant in other age groups.

**Figure 3:**
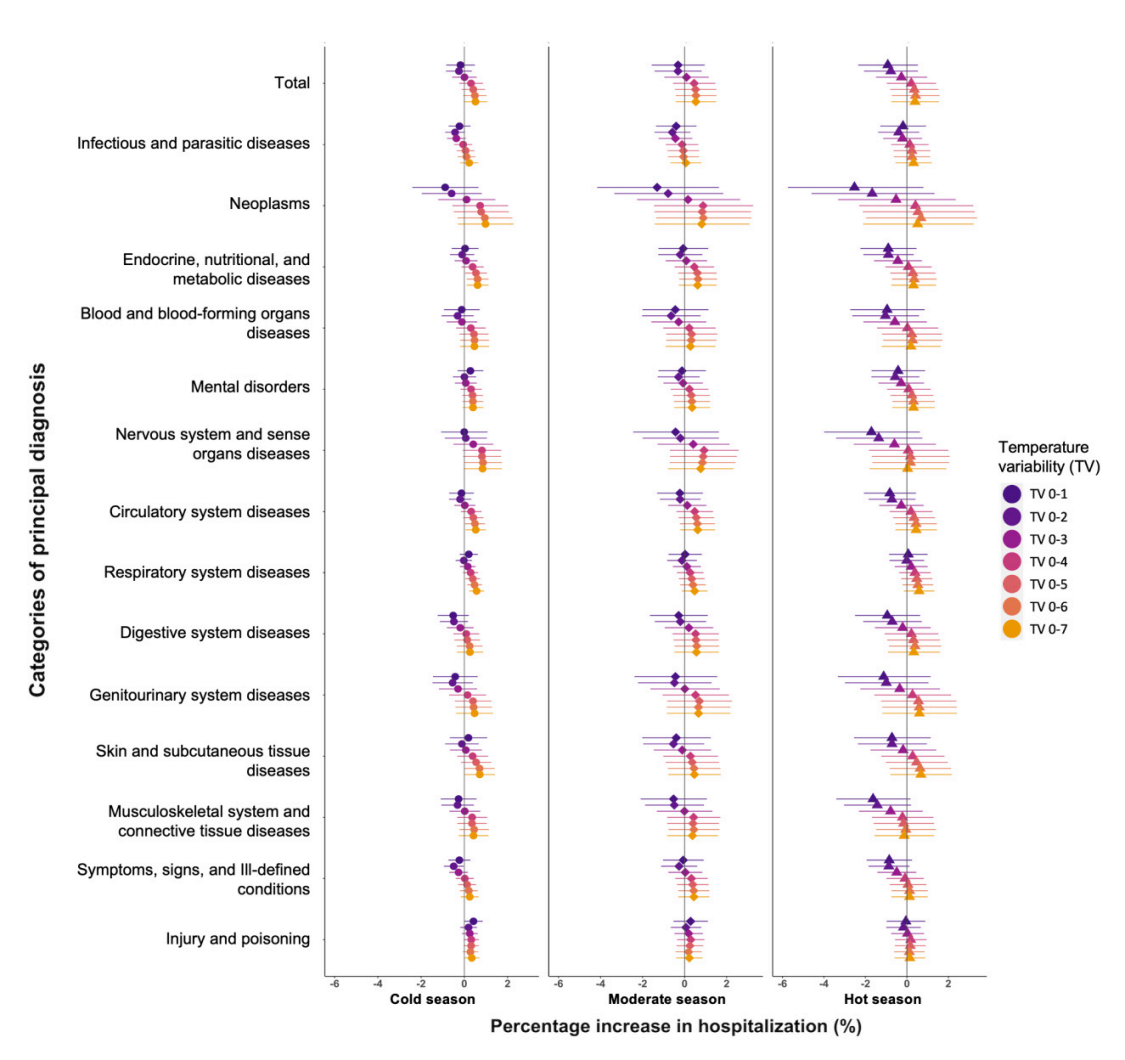
Percentage change (%) in all-cause and cause-specific hospitalizations per interquartile increase in temperature variability (°C) in exposure days from 0-1 to 0-7 days by seasons in Hong Kong. The points in different shapes draw the estimates with 95% CI of the associations in cold seasons (in shape circle), moderate seasons (in shape diamond), hot seasons (in shape triangle)

We found hospitalisation risks associated with TV to be higher during the cold season than in the hot and moderate seasons (**Figure 3**). The admissions increased significantly in endocrine, nutritional, and metabolic diseases (0.54%-0.61%, TV_0-5_ to TV_0-7_), respiratory disease (0.39%-0.57%, TV_0-5_ to TV_0-7_), circulatory system disease (0.49%-0.53%, TV_0-6_ to TV_0-7_), skin and subcutaneous tissue diseases (0.70%-0.71%, TV_0-6_ to TV_0-7_) in cold seasons, which was associated with per IQR unit increase in TV exposures (Table S4 in the **Online Supplementary Document**). We found magnitudes of TV effects to be comparable in moderate and hot seasons while relatively weaker in hot seasons.

**Figure 4:**
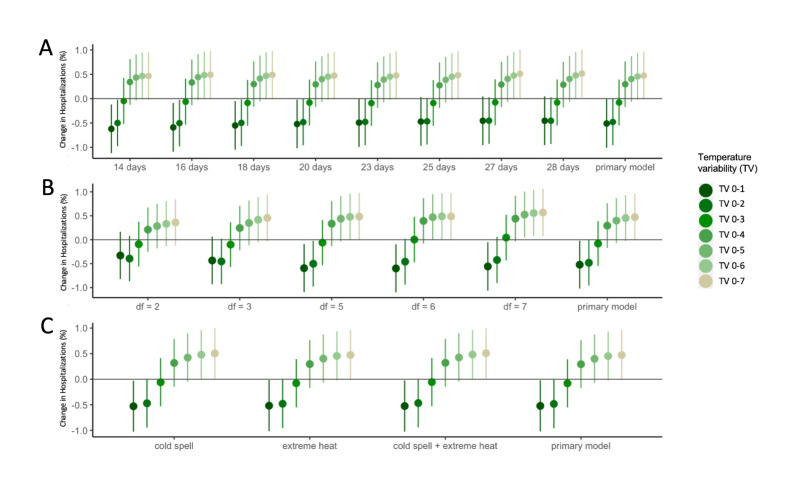
Sensitivity analyses for effect estimates of temperature variability (°C) in exposure days from 0-1 to 0-7 days on all-cause hospitalisations in Hong Kong. **Panel A.** Models with the maximum lag of daily mean temperature at 14-28 days (21 days in primary model). **Panel B.** Models with the degree of freedom (*df*) of daily mean temperature ranged 2-7 (four in primary model). **Panel C.** Models adjusted for cold spells and extreme heat.

Our findings remained robust after altering the maximum lag from 14 to 28 days for *T_mean_* and the degree of freedom from two to seven in smooth functions of *T_mean_* (**Figure 4**). We only observed slight changes occurred in association estimates at TV_0-1_ to TV_0-3_, indicating the insensitive responses to model modifications. The estimates remained consistent after including the cold spell and extreme heat in the model.

## DISCUSSION

Based on 21 years of population-based admissions records in a subtropical setting, we found that TVs are significantly associated with increased risks in hospitalisations for endocrine, nutritional, and metabolic diseases, respiratory system diseases, and circulatory systems diseases. The possible physiological mechanisms of the connection between TVs and human health are not yet fully understood. Current evidence indicates that sudden changes in temperature may activate the sympathetic nervous system, causing responses in body functions, such as the narrowing of blood vessels and increases in heart rate and oxygen consumption [24,25]. Moreover, when experiencing extreme and unstable temperatures, the human automatic thermoregulation system’s inefficient adaptation may enforce the disturbance in blood pressure and blood cholesterol, as well as increase inflammation [26,27]. These impacts would worsen health conditions, particularly for individuals with cardiovascular and respiratory diseases.

We found significant disparities in TV-hospitalisation associations between TV exposures (TV_0-1_ to TV_0-7_) and subgroups by diseases, age, and seasons, with more pronounced health burden associated with TV_0-5_ to TV_0-7_ in people aged ≥50 years and population in cold seasons. The results are mostly in line with previous studies. For example, several multi-country and multi-city studies emphasised the regional heterogeneity in distributions of TV-associated risks across different TV exposures (TV_0-1_ to TV_0-7_) [6,28]. We observed higher effect estimates associated with TV_0-5_ to TV_0-7_, indicating that TVs in longer exposure days are more detrimental to health in Hong Kong, with similar findings reported in Japan, China, the United States, and Australia and converse ones in Brazil and Thailand [6,14,28-30]. Our age-specific analysis showed that the elderly are more vulnerable to ambient TV in all-cause hospitalisation, particularly for those aged over 65 years, which is in line with other studies [28,31]. However, this age-specific susceptibility is not universally applicable to all regions, such as Brazil, where children experienced stronger effects of TV than the elderly [14].

The hospitalisation risks associated with TV in respiratory system disease and circulatory system diseases (hypertensive diseases, cardiovascular diseases, etc.) are corroborated by previous studies in many countries, such as China, Japan, Australia, and Brazil [9,14,29,32,33]. Short-term TVs (TV_0-1_) have a greater impact on respiratory risks in people aged ≤18 years compared to long-term TVs, while the elderly are more susceptible to TV in longer exposure days. The age-related variation in this association could be attributed to the different common respiratory diseases in children and the elderly. Specifically, respiratory diseases with high incidence in children, such as rhinitis, sinusitis, and bronchitis, can be quickly triggered due to their direct exposure to external air and temperature, while the outside environment needs more complex mechanisms to act on organs affected by ageing-associated respiratory diseases, which are generally disorders or injuries in the lungs (eg, asthma, emphysema, and chronic obstructive pulmonary disease).

The elderly have declining physical function due to ageing, including impaired thermoregulatory ability and higher risks of chronic diseases and other associated illnesses, making them more vulnerable to TV exposure [34,35]. We found that almost all the significant TV-associated risks in hospitalisation occurred in people aged ≥50 years. Injury and poisoning in elderly aged ≥65 years were affected most by TVs, and to a substantially greater extent than other diseases. Common accidental injuries in the elderly, such as fractures usually caused by skeletal fragility and falls, increase with age due to weakened muscles and balance control [36]. Potential TV-related risks to fractures generally arise through multiple confounding factors that affect the behaviours of elder people; for example, large temperature changes in spring in Hong Kong and high humidity may lead to the formation of slippery surfaces at their homes.

Notably, we found TV-susceptibility in endocrine, nutritional, and metabolic diseases, and respiratory diseases to be higher in the 50-64 than in the ≥65-year-old elderly group. This could possibly be related to different behaviour adaptations and lifestyles between these two groups. Middle-aged people usually do more outdoor activities and are thus more frequently exposed to ambient temperature, while people after retirement (≥65 years) tend to receive better protection as they stay indoors longer. This gap in TV-related risks caused by behavioural factors is more substantial among people with endocrine, nutritional, and metabolic diseases, due to their high vulnerability to temperature-induced comorbidities. The endocrine gland and hormonal regulation are closely involved in body thermoregulation, particularly the thyroid. Thus, the disorders and injuries in such organs and functions will directly impact the adaptions to temperature change. For example, people with diabetes are reported to have a weakened capacity to dissipate heat due to lower skin blood flow and sweating responses [37].

People aged ≤18 years experienced the highest TV-associated risks in hospitalization for the nervous system and sense organs diseases. This finding is supported by a scoping review that incidences or visits of neurological disorders (ie, stroke, dementia, headache) are associated with temperature fluctuations; however, literature on this association among children is very limited [38]. Several common diseases of sense organs among children (ie, infections of eyes or ears, conjunctivitis) showed elevated risks when the temperature changed [39,40]. Children's barrier functions in eyes and ears against sunlight, temperature, and bacterial and viral infections are not fully developed as adults. Sudden temperature changes are more likely to trigger colds, fever, and upper respiratory infections among children, as they have weaker immunity and temperature adaption, thus producing a microbe-friendly environment for secondary infections in the ear and eyes. Moreover, children and adolescents may be less aware of controlling their scratching and rubbing, which would exacerbate inflammation and disorders [40,41].

The seasonal differences in TV-associated hospitalisation risks are not substantial in Hong Kong, which may be attributed to Hong Kong's relatively mild weather, except for the heat in summer. TV effects in cold seasons are more substantial than in other seasons, with an additional significantly associated risk of hospitalisation for skin and subcutaneous tissue diseases. Some of the reasons for this finding might be the high air conditioning coverage in Hong Kong, which allows for regulation of indoor temperature and reduces the TV impact during the hot days, while the decreased use of air conditioners during mild winters makes people more likely to be exposed to TVs in cold seasons. Studies have shown a J-shaped exposure-response curve between TV and hospitalisations, with increasing health risks when TV exposures are over the threshold of 3.0°C [9,33]. The seasonal pattern of TVs in Hong Kong illustrated that only the average TVs in cold seasons exceed 3.0°C, thus resulting in stronger associations (Figure S1 in the **Online Supplementary Document**); More frequent outdoor mobilisation and activities during the holiday seasons from December to January may lead to increased direct exposure to external air temperatures.

This study has some limitations. First, we used the meteorological data collected by fixed-site monitoring stations and defined the TV exposure levels by city-wide measurements instead of individual-level exposure, preventing causal inference when investigating TV-hospitalisation associations. Additionally, this approach may introduce exposure misclassification bias since TV exposures can vary significantly within the city, affected by factors such as residential environment and heat island effect. Thus, obtaining more precise measurements of TV exposure is necessary to avoid the potential underestimations of the TV effect [42]. Second, our hospitalisation data lack individual-level information about sex and health risk factors, including obesity, physical inactivity, alcohol intake, and medical history, which contribute to the hospitalisation risks as well. Third, the definitions of TV are inconsistent among the previous studies, as the weighted standard deviation of *T_min_* and *T_max_* in current and following N days for calculating TV_0-N_ is accepted [43].

## CONCLUSIONS

We found that the associations between TV and hospitalisations varied between TV exposure days, age groups, and diseases in Hong Kong, a subtropical setting in Asia. Generally, we observed increases in TV_0-5_ to TV_0-7_ exposures to be associated with detrimental effects on hospitalisations in Hong Kong and people aged ≥50 years to be more vulnerable to the TV exposures. Hospitalisations for endocrine, nutritional and metabolic diseases, respiratory, and circulatory systems diseases were associated with TV exposures. The health burden related to TVs, usually regarded as minimal compared to other environmental factors, should receive more attention, especially for the vulnerable population during the cold seasons. Our study provides more detailed information for shaping the relevant health policies about climate change in Hong Kong, delineating the profiles of potential high-risk populations, which may contribute to enhancing the precision and adaptability of strategies, such as public awareness campaigns, temperature control in public spaces, and health care system preparedness.

## Additional material


Online Supplementary Document
