# Supplementary material for: Relationship between temperature variability and daily hospitalisations in Hong Kong over two decades
Source: J Glob Health. 2023 Oct 13;13:04122. doi: 10.7189/jogh.13.04122 (PMC10569366; doi:10.7189/jogh.13.04122)

Relationship between temperature variability and daily hospitalizations in Hong Kong over two decades

Kehang Li, Yawen Wang, Xiaoting Jiang, Conglu Li, Jinjian Chen, Yiqian Zeng, Shi Zhao, Janice Ho, Jinjun Ran, Lefei Han, Yuchen Wei, Eng Kiong Yeoh, Ka Chun Chong

Online Supplementary Document

Table S1: Classification of hospital admissions based on International Classification of Diseases, Ninth Revision (ICD-9-CM)

Table S2: Summaries of total hospital admissions and average daily admissions by causes of admission, age groups, and seasons in Hong Kong from 1999 to 2019.

Table S3: Age-specific percentage change in hospitalizations by causes associated with per interquartile increase in temperature variability (°C) in exposure days from 0–1 to 0–7 days in Hong Kong.

Table S4: Season-specific percentage change in hospitalizations by causes associated with per interquartile increase in temperature variability (°C) in exposure days from 0–1 to 0–7 days in Hong Kong.

Figure S1: Monthly trends from January to December of average temperature variabilities (°C) in exposure days from 0–1 to 0–7 days during 1999 to 2019 in Hong Kong.

Table S1. Classification of hospital admissions based on International Classification of Diseases, Ninth Revision (ICD-9-CM)

No.	Principal diagnosis	ICD-9-CM
1	Infectious and parasitic diseases	001.xx - 139.xx
2	Neoplasms	140.xx – 239.xx
3	Endocrine, nutritional, and metabolic diseases	240.xx – 279.xx
4	Blood and blood-forming organs diseases	280.xx – 289.xx
5	Mental disorders	290.xx – 319.xx
6	Nervous system and sense organs diseases	320.xx – 389.xx
7	Circulatory system diseases	390.xx – 459.xx
8	Respiratory system diseases	460.xx – 519.xx
9	Digestive system diseases	520.xx – 579.xx
10	Genitourinary system diseases	580.xx – 629.xx
11	Skin and subcutaneous tissue diseases	680.xx – 709.xx
12	Musculoskeletal system and connective tissue diseases	710.xx – 739.xx
13	Symptoms, signs, and Ill-defined conditions	780.xx – 799.xx
14	Injury and poisoning	800.xx – 999.xx

Table S2. Summaries of total hospital admissions and average daily admissions by causes of admission, age groups, and seasons in Hong Kong from 1999 to 2019.

Age group	Total admissions	Average daily admissions			
	N (%)	Total	Cold seasons	Hot seasons	Moderate seasons
**All-cause**					
0 – 18 years	2955748 (7.76%)	385.36	390.38	383.37	383.98
19 – 49 years	6802655 (17.86%)	886.91	850.47	910.03	889.97
50 - 64 years	7943881 (20.85%)	1035.70	1011.99	1049.84	1038.41
65 - 79 years	10904143 (28.63%)	1421.66	1447.15	1404.95	1419.94
≥ 80 years	9525633 (25.01%)	1241.93	1343.78	1183.10	1228.77
Total	38091114 (100%)	4966.24	5038.56	4927.45	4954.52
**Infectious and parasitic diseases**					
0 – 18 years	422477 (26.43%)	55.08	57.67	55.35	53.34
19 – 49 years	301027 (18.83%)	39.25	38.21	40.17	39.12
50 - 64 years	218555 (13.67%)	28.50	27.81	29.14	28.39
65 - 79 years	316106 (19.78%)	41.21	41.34	41.57	40.85
≥ 80 years	340270 (21.29%)	44.36	46.16	43.97	43.62
Total	1598344 (100%)	208.42	211.19	210.20	205.36
**Neoplasms**					
0 – 18 years	166961 (4.42%)	21.80	21.73	22.60	21.20
19 – 49 years	737004 (19.51%)	96.09	92.83	98.03	96.46
50 - 64 years	1266598 (33.53%)	165.14	159.44	168.47	165.84
65 - 79 years	1154168 (30.56%)	150.48	145.71	153.43	150.94
≥ 80 years	453987 (12.02%)	59.19	58.89	59.41	59.19
Total	3777316 (100%)	493.19	478.94	502.66	494.04
**Endocrine, nutritional, and metabolic diseases**					
0 – 18 years	109797 (3.61%)	14.37	15.46	14.42	13.68
19 – 49 years	312569 (10.27%)	40.75	39.58	41.58	40.78
50 - 64 years	595902 (19.58%)	77.69	77.08	77.86	77.92
65 - 79 years	1069546 (35.13%)	139.45	144.54	135.97	139.21
≥ 80 years	960858 (31.56%)	125.27	135.78	118.56	124.43
Total	3044177 (100%)	398.35	413.48	389.12	396.79
**Blood and blood-forming organs diseases**					
0 – 18 years	140460 (10.61%)	18.31	18.36	18.18	18.39
19 – 49 years	341498 (25.8%)	44.52	43.35	45.32	44.59
50 - 64 years	179584 (13.57%)	23.45	23.04	23.58	23.58
65 - 79 years	294433 (22.24%)	38.39	38.90	38.06	38.34
≥ 80 years	368493 (27.83%)	48.04	51.52	46.17	47.49
Total	1323853 (100%)	172.85	175.27	171.50	172.50
**Mental disorders**					
0 – 18 years	74424 (5.73%)	9.75	9.42	9.31	10.29
19 – 49 years	393025 (30.27%)	51.24	47.97	52.73	51.98
50 - 64 years	187886 (14.47%)	24.50	23.28	25.19	24.66
65 - 79 years	231943 (17.86%)	30.24	30.28	30.47	30.03
≥ 80 years	413696 (31.86%)	53.94	57.16	52.13	53.48
Total	1298326 (100%)	170.00	168.68	170.13	170.68
**Nervous system and sense organs diseases**					
0 – 18 years	170998 (9.13%)	22.29	22.15	22.52	22.20
19 – 49 years	258775 (13.81%)	33.74	32.41	34.43	33.97
50 - 64 years	372901 (19.9%)	48.62	47.47	49.27	48.77
65 - 79 years	628116 (33.52%)	81.89	80.37	83.83	81.24
≥ 80 years	442815 (23.63%)	57.73	58.82	57.95	56.92
Total	1873605 (100%)	244.28	241.22	248.01	243.10
**Circulatory system diseases**					
0 – 18 years	70863 (1.43%)	9.33	9.20	9.62	9.18
19 – 49 years	410606 (8.31%)	53.53	52.75	54.02	53.61
50 - 64 years	950799 (19.25%)	123.96	123.55	123.60	124.50
65 - 79 years	1791253 (36.27%)	233.54	245.56	224.36	233.77

≥ 80 years	1743228 (35.3%)	227.28	254.92	209.41	225.22
Total	4938349 (100%)	650.30	688.70	622.79	649.64
**Respiratory system diseases**					
0 – 18 years	637539 (17.65%)	83.12	85.74	78.08	85.59
19 – 49 years	313180 (8.67%)	40.83	41.97	40.13	40.72
50 - 64 years	399134 (11.05%)	52.04	55.37	49.65	51.98
65 - 79 years	1006898 (27.87%)	131.28	145.49	121.44	130.74
≥ 80 years	1255564 (34.76%)	163.70	190.22	147.42	161.04
Total	3612315 (100%)	470.97	518.80	436.71	470.07
**Digestive system diseases**					
0 – 18 years	198356 (7.05%)	25.86	28.15	24.92	25.26
19 – 49 years	500085 (17.78%)	65.20	63.33	66.58	65.20
50 - 64 years	623420 (22.16%)	81.28	79.04	82.30	81.79
65 - 79 years	857746 (30.49%)	111.83	111.69	111.79	111.95
≥ 80 years	633731 (22.53%)	82.62	86.69	80.50	81.92
Total	2813338 (100%)	366.80	368.91	366.09	366.12
**Genitourinary system diseases**					
0 – 18 years	175234 (3.33%)	22.85	21.68	25.81	21.17
19 – 49 years	1282084 (24.37%)	167.16	159.61	170.93	168.60
50 - 64 years	1466326 (27.87%)	191.18	188.01	193.22	191.41
65 - 79 years	1435551 (27.29%)	187.16	187.86	186.67	187.15
≥ 80 years	901968 (17.14%)	117.60	122.56	114.85	116.86
Total	5261163 (100%)	685.94	679.72	691.47	685.20
**Skin and subcutaneous tissue diseases**					
0 – 18 years	90936 (11.41%)	11.86	11.53	12.35	11.67
19 – 49 years	189771 (23.81%)	24.74	23.07	26.33	24.46
50 - 64 years	150584 (18.89%)	19.65	17.97	21.12	19.46
65 - 79 years	170602 (21.4%)	22.25	21.28	23.31	21.97
≥ 80 years	195606 (24.54%)	25.51	25.93	25.15	25.55
Total	797141 (100%)	104.08	99.83	108.29	103.22
**Musculoskeletal system and connective tissue diseases**					
0 – 18 years	61646 (4.77%)	8.07	7.98	8.60	7.70
19 – 49 years	291904 (22.61%)	38.06	35.97	39.20	38.38
50 - 64 years	295600 (22.9%)	38.54	35.97	39.95	38.95
65 - 79 years	350859 (27.18%)	45.74	45.28	45.59	46.14
≥ 80 years	294023 (22.77%)	38.33	43.57	35.22	37.73
Total	1291095 (100%)	169.08	169.16	168.82	169.24
**Symptoms, signs, and Ill-defined conditions**					
0 – 18 years	392364 (8.66%)	51.16	51.70	50.68	51.22
19 – 49 years	937075 (20.68%)	122.17	114.40	127.43	122.57
50 - 64 years	883122 (19.49%)	115.14	109.82	118.20	115.84
65 - 79 years	1212058 (26.74%)	158.03	156.91	158.64	158.20
≥ 80 years	1107402 (24.44%)	144.38	149.49	142.28	143.04
Total	4532021 (100%)	590.88	582.32	597.22	590.87
**Injury and poisoning**					
0 – 18 years	243693 (12.63%)	31.77	29.89	31.17	33.36
19 – 49 years	534052 (27.67%)	69.63	65.01	73.15	69.54
50 - 64 years	353470 (18.31%)	46.08	44.18	48.37	45.39
65 - 79 years	384864 (19.94%)	50.18	51.96	49.81	49.42
≥ 80 years	413992 (21.45%)	53.98	62.08	50.10	52.28
Total	1930071 (100%)	251.64	253.12	252.61	249.99

Cold seasons: months from December to February in next year
Hot seasons: months from July to September
Moderate seasons: months from March to May, October to November

Table S3. Age-specific percentage change in hospitalizations by causes associated with per interquartile increase in temperature variability (°C) in exposure days from 0–1 to 0–7 days in Hong Kong.

TV exposure	Percentage change in hospitalization (%) (95% CI)					
	**Total**	**0–18 years**	**19–49 years**	**50–64 years**	**65–79 years**	**≥ 80 years**
**All-cause hospitalization**						
TV_{0-1} (°C)	-0.41 (-0.92, 0.09)	0.07 (-0.38, 0.52)	-0.61 (-1.22, 0.02)	-0.76 (-1.48, -0.04)	-0.43 (-0.97, 0.12)	-0.20 (-0.54, 0.15)
TV_{0-2} (°C)	-0.39 (-0.87, 0.09)	-0.17 (-0.60, 0.26)	-0.54 (-1.12, 0.05)	-0.58 (-1.27, 0.11)	-0.39 (-0.91, 0.13)	-0.32 (-0.65, 0.01)
TV_{0-3} (°C)	-0.01 (-0.48, 0.47)	-0.01 (-0.43, 0.41)	-0.16 (-0.74, 0.42)	0.00 (-0.67, 0.68)	0.02 (-0.49, 0.53)	-0.04 (-0.36, 0.28)
TV_{0-4} (°C)	0.36 (-0.11, 0.83)	0.21 (-0.21, 0.63)	0.21 (-0.37, 0.78)	0.53 (-0.14, 1.21)	0.38 (-0.13, 0.89)	0.30 (-0.02, 0.61)
TV_{0-5} (°C)	0.45 (-0.02, 0.93)	0.21 (-0.21, 0.63)	0.17 (-0.41, 0.75)	0.67 (-0.01, 1.35)	0.46 (-0.05, 0.97)	0.48 (0.16, 0.80)
TV_{0-6} (°C)	0.50 (0.02, 0.98)	0.23 (-0.19, 0.66)	0.12 (-0.47, 0.71)	0.74 (0.05, 1.43)	0.53 (0.02, 1.05)	0.55 (0.23, 0.88)
TV_{0-7} (°C)	0.51 (0.02, 1.00)	0.27 (-0.16, 0.71)	0.09 (-0.51, 0.70)	0.71 (0.00, 1.42)	0.57 (0.04, 1.10)	0.61 (0.28, 0.94)
**Infectious and parasitic diseases**						
TV_{0-1} (°C)	-0.30 (-0.69, 0.09)	0.00 (-0.56, 0.56)	-0.39 (-1.18, 0.40)	-1.31 (-2.11, -0.50)	-0.09 (-0.74, 0.56)	-0.16 (-0.75, 0.44)
TV_{0-2} (°C)	-0.49 (-0.86, -0.13)	-0.11 (-0.64, 0.42)	-0.59 (-1.34, 0.16)	-1.05 (-1.81, -0.28)	-0.4 (-1.02, 0.21)	-0.6 (-1.16, -0.03)
TV_{0-3} (°C)	-0.37 (-0.73, -0.01)	-0.21 (-0.73, 0.31)	-0.34 (-1.08, 0.40)	-0.60 (-1.36, 0.15)	-0.39 (-0.99, 0.22)	-0.37 (-0.92, 0.18)
TV_{0-4} (°C)	-0.05 (-0.41, 0.31)	-0.23 (-0.74, 0.29)	0.15 (-0.58, 0.89)	0.15 (-0.60, 0.91)	-0.16 (-0.75, 0.44)	0.03 (-0.52, 0.58)
TV_{0-5} (°C)	0.03 (-0.33, 0.39)	-0.32 (-0.84, 0.20)	0.11 (-0.63, 0.85)	0.42 (-0.34, 1.18)	-0.07 (-0.67, 0.54)	0.32 (-0.23, 0.88)
TV_{0-6} (°C)	0.05 (-0.31, 0.42)	-0.39 (-0.92, 0.14)	-0.07 (-0.82, 0.68)	0.53 (-0.24, 1.30)	0.10 (-0.51, 0.71)	0.44 (-0.12, 1.00)
TV_{0-7} (°C)	0.17 (-0.21, 0.54)	-0.30 (-0.84, 0.24)	-0.09 (-0.86, 0.68)	0.64 (-0.15, 1.43)	0.32 (-0.30, 0.95)	0.59 (0.02, 1.16)
**Neoplasms**						
TV_{0-1} (°C)	-1.48 (-2.63, -0.31)	-0.21 (-1.66, 1.26)	-1.52 (-2.85, -0.18)	-1.68 (-3.01, -0.33)	-1.54 (-2.68, -0.40)	-1.18 (-2.01, -0.35)
TV_{0-2} (°C)	-0.92 (-2.02, 0.20)	-0.30 (-1.67, 1.09)	-0.87 (-2.14, 0.41)	-0.98 (-2.26, 0.31)	-0.96 (-2.04, 0.14)	-0.97 (-1.75, -0.18)
TV_{0-3} (°C)	0.01 (-1.09, 1.11)	0.57 (-0.78, 1.94)	0.06 (-1.20, 1.32)	0.08 (-1.19, 1.36)	-0.05 (-1.12, 1.04)	-0.32 (-1.10, 0.46)
TV_{0-4} (°C)	0.74 (-0.35, 1.84)	1.35 (0.00, 2.73)	0.65 (-0.60, 1.92)	1.05 (-0.22, 2.34)	0.62 (-0.45, 1.71)	0.16 (-0.61, 0.93)
TV_{0-5} (°C)	0.76 (-0.34, 1.88)	1.10 (-0.27, 2.48)	0.55 (-0.72, 1.83)	1.18 (-0.11, 2.48)	0.63 (-0.46, 1.73)	0.29 (-0.49, 1.08)
TV_{0-6} (°C)	0.88 (-0.24, 2.01)	0.95 (-0.44, 2.36)	0.58 (-0.70, 1.89)	1.37 (0.06, 2.69)	0.73 (-0.38, 1.84)	0.44 (-0.35, 1.24)
TV_{0-7} (°C)	0.84 (-0.30, 2.00)	0.89 (-0.53, 2.33)	0.62 (-0.70, 1.95)	1.30 (-0.04, 2.65)	0.61 (-0.52, 1.76)	0.57 (-0.24, 1.39)
**Endocrine, nutritional, and metabolic diseases**						
TV_{0-1} (°C)	-0.23 (-0.70, 0.25)	0.05 (-1.23, 1.35)	-0.60 (-1.52, 0.33)	-0.52 (-1.25, 0.21)	0.01 (-0.51, 0.53)	-0.24 (-0.66, 0.18)
TV_{0-2} (°C)	-0.32 (-0.77, 0.14)	-0.20 (-1.42, 1.02)	-0.81 (-1.68, 0.07)	-0.40 (-1.10, 0.29)	-0.18 (-0.67, 0.31)	-0.26 (-0.65, 0.14)
TV_{0-3} (°C)	-0.02 (-0.46, 0.43)	-0.14 (-1.32, 1.06)	-0.34 (-1.20, 0.52)	0.17 (-0.51, 0.86)	0.09 (-0.39, 0.57)	-0.07 (-0.46, 0.32)
TV_{0-4} (°C)	0.37 (-0.07, 0.81)	0.05 (-1.12, 1.24)	0.20 (-0.65, 1.06)	0.72 (0.05, 1.41)	0.37 (-0.10, 0.85)	0.29 (-0.09, 0.68)
TV_{0-5} (°C)	0.53 (0.09, 0.97)	0.11 (-1.07, 1.31)	0.25 (-0.61, 1.12)	0.89 (0.21, 1.58)	0.51 (0.03, 0.99)	0.55 (0.16, 0.94)
TV_{0-6} (°C)	0.59 (0.14, 1.04)	0.06 (-1.15, 1.27)	0.08 (-0.79, 0.96)	0.94 (0.25, 1.63)	0.65 (0.16, 1.13)	0.61 (0.22, 1.01)
TV_{0-7} (°C)	0.58 (0.12, 1.03)	-0.12 (-1.35, 1.12)	-0.16 (-1.05, 0.73)	0.80 (0.10, 1.51)	0.69 (0.20, 1.19)	0.69 (0.29, 1.09)
**Blood and blood-forming organs diseases**						
TV_{0-1} (°C)	-0.47 (-1.10, 0.16)	-0.05 (-1.29, 1.21)	-0.71 (-1.57, 0.15)	-0.98 (-2.00, 0.06)	-0.41 (-1.23, 0.42)	-0.29 (-0.95, 0.37)
TV_{0-2} (°C)	-0.62 (-1.22, -0.02)	-0.42 (-1.6, 0.77)	-0.72 (-1.53, 0.10)	-0.92 (-1.90, 0.07)	-0.65 (-1.43, 0.13)	-0.49 (-1.11, 0.14)
TV_{0-3} (°C)	-0.28 (-0.86, 0.31)	-0.29 (-1.44, 0.87)	-0.27 (-1.07, 0.54)	-0.44 (-1.40, 0.53)	-0.36 (-1.12, 0.40)	-0.21 (-0.82, 0.41)
TV_{0-4} (°C)	0.22 (-0.36, 0.80)	0.52 (-0.63, 1.68)	0.31 (-0.49, 1.11)	0.35 (-0.60, 1.32)	0.04 (-0.72, 0.80)	0.05 (-0.55, 0.66)
TV_{0-5} (°C)	0.36 (-0.22, 0.95)	0.53 (-0.62, 1.70)	0.37 (-0.43, 1.18)	0.66 (-0.30, 1.64)	0.15 (-0.61, 0.92)	0.27 (-0.34, 0.88)
TV_{0-6} (°C)	0.38 (-0.22, 0.97)	0.27 (-0.90, 1.46)	0.09 (-0.72, 0.91)	0.91 (-0.07, 1.90)	0.27 (-0.50, 1.04)	0.46 (-0.16, 1.08)
TV_{0-7} (°C)	0.35 (-0.25, 0.96)	0.33 (-0.87, 1.54)	-0.18 (-1.01, 0.65)	0.93 (-0.07, 1.94)	0.39 (-0.39, 1.18)	0.49 (-0.14, 1.12)
**Mental disorders**						
TV_{0-1} (°C)	-0.08 (-0.53, 0.37)	-0.6 (-2.04, 0.86)	-0.14 (-0.77, 0.48)	-0.51 (-1.34, 0.32)	0.43 (-0.30, 1.16)	-0.17 (-0.72, 0.38)
TV_{0-2} (°C)	-0.25 (-0.68, 0.17)	-0.13 (-1.50, 1.26)	-0.09 (-0.69, 0.51)	-0.56 (-1.35, 0.23)	0.14 (-0.55, 0.83)	-0.58 (-1.10, -0.05)
TV_{0-3} (°C)	-0.06 (-0.48, 0.36)	0.29 (-1.05, 1.65)	0.02 (-0.56, 0.61)	-0.17 (-0.94, 0.61)	0.25 (-0.43, 0.93)	-0.34 (-0.85, 0.18)
TV_{0-4} (°C)	0.24 (-0.18, 0.65)	0.37 (-0.96, 1.73)	0.05 (-0.52, 0.64)	0.04 (-0.73, 0.81)	0.61 (-0.06, 1.28)	0.27 (-0.24, 0.78)
TV_{0-5} (°C)	0.33 (-0.09, 0.75)	0.34 (-1.01, 1.71)	-0.01 (-0.60, 0.57)	0.15 (-0.63, 0.93)	0.65 (-0.02, 1.33)	0.55 (0.05, 1.06)
TV_{0-6} (°C)	0.36 (-0.06, 0.79)	0.35 (-1.02, 1.74)	0.02 (-0.58, 0.61)	0.08 (-0.70, 0.88)	0.68 (0.00, 1.37)	0.65 (0.13, 1.16)
TV_{0-7} (°C)	0.37 (-0.06, 0.81)	0.64 (-0.76, 2.06)	-0.02 (-0.63, 0.59)	-0.01 (-0.81, 0.80)	0.74 (0.04, 1.44)	0.68 (0.15, 1.20)
**Nervous system and sense organs diseases**						
TV_{0-1} (°C)	-0.61 (-1.43, 0.21)	0.00 (-1.11, 1.13)	-0.44 (-1.35, 0.48)	-0.81 (-1.81, 0.20)	-0.74 (-1.71, 0.24)	-0.61 (-1.42, 0.22)
TV_{0-2} (°C)	-0.37 (-1.14, 0.41)	-0.18 (-1.23, 0.88)	0.10 (-0.76, 0.98)	-0.62 (-1.58, 0.34)	-0.39 (-1.31, 0.54)	-0.45 (-1.23, 0.33)
TV_{0-3} (°C)	0.21 (-0.55, 0.98)	0.47 (-0.56, 1.51)	0.45 (-0.40, 1.30)	0.04 (-0.90, 0.99)	0.24 (-0.67, 1.15)	0.10 (-0.66, 0.87)
TV_{0-4} (°C)	0.73 (-0.03, 1.49)	1.04 (0.01, 2.07)	0.87 (0.03, 1.72)	0.51 (-0.43, 1.45)	0.89 (-0.02, 1.80)	0.51 (-0.25, 1.27)
TV_{0-5} (°C)	0.74 (-0.02, 1.51)	1.02 (-0.02, 2.06)	0.70 (-0.15, 1.56)	0.57 (-0.37, 1.53)	0.91 (0.00, 1.83)	0.57 (-0.19, 1.34)
TV_{0-6} (°C)	0.75 (-0.02, 1.53)	1.15 (0.09, 2.21)	0.50 (-0.36, 1.37)	0.72 (-0.24, 1.69)	0.91 (-0.01, 1.85)	0.56 (-0.22, 1.34)
TV_{0-7} (°C)	0.70 (-0.10, 1.49)	1.15 (0.07, 2.24)	0.55 (-0.34, 1.44)	0.72 (-0.25, 1.71)	0.82 (-0.13, 1.77)	0.43 (-0.36, 1.23)
**Circulatory system diseases**						
TV_{0-1} (°C)	-0.33 (-0.77, 0.11)	-0.41 (-1.90, 1.10)	-0.66 (-1.46, 0.15)	-0.64 (-1.30, 0.04)	-0.20 (-0.69, 0.28)	-0.17 (-0.53, 0.20)
TV_{0-2} (°C)	-0.31 (-0.73, 0.11)	-0.45 (-1.87, 0.98)	-0.65 (-1.41, 0.12)	-0.44 (-1.07, 0.20)	-0.19 (-0.65, 0.27)	-0.2 (-0.55, 0.15)
TV_{0-3} (°C)	0.02 (-0.39, 0.43)	0.26 (-1.14, 1.67)	-0.27 (-1.02, 0.48)	0.03 (-0.60, 0.65)	0.14 (-0.31, 0.59)	0.01 (-0.33, 0.35)
TV_{0-4} (°C)	0.37 (-0.03, 0.78)	0.67 (-0.72, 2.08)	0.19 (-0.55, 0.94)	0.38 (-0.24, 1.00)	0.43 (-0.02, 0.87)	0.34 (0.01, 0.68)
TV_{0-5} (°C)	0.47 (0.06, 0.88)	1.05 (-0.36, 2.47)	0.03 (-0.72, 0.78)	0.43 (-0.20, 1.05)	0.51 (0.06, 0.95)	0.52 (0.19, 0.86)

TV_{0-6} (°C)	0.53 (0.12, 0.95)	1.40 (-0.04, 2.85)	-0.17 (-0.93, 0.59)	0.46 (-0.17, 1.09)	0.61 (0.15, 1.06)	0.59 (0.25, 0.94)
TV_{0-7} (°C)	0.56 (0.14, 0.98)	1.21 (-0.26, 2.69)	-0.26 (-1.04, 0.52)	0.35 (-0.29, 1.00)	0.69 (0.23, 1.16)	0.66 (0.31, 1.00)
**Respiratory system diseases**						
TV_{0-1} (°C)	0.09 (-0.24, 0.41)	0.42 (-0.18, 1.02)	-0.29 (-1.04, 0.46)	0.09 (-0.54, 0.73)	-0.14 (-0.58, 0.30)	0.18 (-0.18, 0.54)
TV_{0-2} (°C)	-0.07 (-0.38, 0.24)	-0.04 (-0.61, 0.53)	-0.08 (-0.79, 0.64)	0.18 (-0.42, 0.78)	-0.14 (-0.56, 0.28)	-0.11 (-0.45, 0.24)
TV_{0-3} (°C)	0.14 (-0.16, 0.44)	0.03 (-0.53, 0.59)	0.46 (-0.24, 1.16)	0.38 (-0.21, 0.98)	0.06 (-0.35, 0.47)	0.11 (-0.23, 0.44)
TV_{0-4} (°C)	0.29 (0.00, 0.59)	0.14 (-0.41, 0.69)	0.45 (-0.24, 1.14)	0.59 (0.01, 1.18)	0.14 (-0.27, 0.54)	0.37 (0.04, 0.70)
TV_{0-5} (°C)	0.38 (0.08, 0.68)	0.15 (-0.40, 0.71)	0.37 (-0.33, 1.08)	0.73 (0.14, 1.32)	0.20 (-0.21, 0.61)	0.52 (0.19, 0.86)
TV_{0-6} (°C)	0.45 (0.14, 0.75)	0.27 (-0.29, 0.84)	0.39 (-0.32, 1.10)	0.83 (0.24, 1.43)	0.27 (-0.14, 0.68)	0.56 (0.22, 0.90)
TV_{0-7} (°C)	0.53 (0.22, 0.84)	0.42 (-0.16, 1.00)	0.46 (-0.26, 1.19)	0.85 (0.24, 1.46)	0.39 (-0.03, 0.82)	0.60 (0.26, 0.95)
**Digestive system diseases**						
TV_{0-1} (°C)	-0.50 (-1.06, 0.05)	0.29 (-0.60, 1.19)	-0.8 (-1.52, -0.07)	-0.59 (-1.39, 0.22)	-0.62 (-1.28, 0.05)	-0.25 (-0.74, 0.23)
TV_{0-2} (°C)	-0.40 (-0.92, 0.13)	0.22 (-0.62, 1.07)	-0.83 (-1.51, -0.13)	-0.27 (-1.03, 0.50)	-0.48 (-1.11, 0.15)	-0.25 (-0.71, 0.21)
TV_{0-3} (°C)	-0.01 (-0.53, 0.5)	0.46 (-0.36, 1.29)	-0.46 (-1.14, 0.22)	0.11 (-0.64, 0.87)	0.03 (-0.59, 0.66)	0.05 (-0.40, 0.51)
TV_{0-4} (°C)	0.31 (-0.21, 0.82)	0.57 (-0.25, 1.40)	-0.09 (-0.76, 0.59)	0.48 (-0.27, 1.24)	0.44 (-0.18, 1.05)	0.24 (-0.20, 0.69)
TV_{0-5} (°C)	0.34 (-0.17, 0.86)	0.58 (-0.25, 1.41)	-0.17 (-0.85, 0.51)	0.48 (-0.28, 1.24)	0.62 (-0.01, 1.24)	0.23 (-0.22, 0.68)
TV_{0-6} (°C)	0.41 (-0.11, 0.94)	0.70 (-0.14, 1.55)	-0.18 (-0.87, 0.51)	0.61 (-0.16, 1.38)	0.73 (0.10, 1.37)	0.22 (-0.23, 0.68)
TV_{0-7} (°C)	0.40 (-0.13, 0.94)	0.60 (-0.26, 1.46)	-0.25 (-0.96, 0.46)	0.61 (-0.18, 1.40)	0.73 (0.08, 1.38)	0.26 (-0.20, 0.73)
**Genitourinary system diseases**						
TV_{0-1} (°C)	-0.59 (-1.38, 0.20)	-0.04 (-1.25, 1.18)	-0.73 (-1.66, 0.21)	-0.84 (-1.86, 0.20)	-0.52 (-1.33, 0.30)	-0.25 (-0.74, 0.25)
TV_{0-2} (°C)	-0.61 (-1.36, 0.14)	0.29 (-0.85, 1.45)	-0.45 (-1.34, 0.44)	-0.86 (-1.84, 0.12)	-0.64 (-1.40, 0.14)	-0.54 (-1.01, -0.08)
TV_{0-3} (°C)	-0.16 (-0.89, 0.58)	0.21 (-0.91, 1.35)	-0.01 (-0.87, 0.87)	-0.28 (-1.24, 0.69)	-0.17 (-0.92, 0.59)	-0.22 (-0.67, 0.24)
TV_{0-4} (°C)	0.34 (-0.39, 1.07)	0.39 (-0.73, 1.51)	0.52 (-0.34, 1.40)	0.33 (-0.63, 1.30)	0.33 (-0.42, 1.09)	0.14 (-0.32, 0.59)
TV_{0-5} (°C)	0.56 (-0.18, 1.30)	0.61 (-0.51, 1.76)	0.76 (-0.11, 1.64)	0.63 (-0.34, 1.61)	0.48 (-0.27, 1.24)	0.34 (-0.12, 0.79)
TV_{0-6} (°C)	0.55 (-0.19, 1.31)	0.54 (-0.60, 1.71)	0.74 (-0.15, 1.64)	0.55 (-0.44, 1.54)	0.50 (-0.27, 1.27)	0.45 (-0.01, 0.91)
TV_{0-7} (°C)	0.57 (-0.19, 1.34)	0.35 (-0.83, 1.54)	0.73 (-0.19, 1.65)	0.55 (-0.46, 1.57)	0.53 (-0.25, 1.33)	0.55 (0.08, 1.03)
**Skin and subcutaneous tissue diseases**						
TV_{0-1} (°C)	-0.31 (-0.96, 0.34)	0.01 (-1.20, 1.24)	-0.91 (-1.92, 0.11)	0.18 (-0.87, 1.23)	-0.01 (-0.93, 0.90)	-0.45 (-1.24, 0.35)
TV_{0-2} (°C)	-0.44 (-1.06, 0.18)	0.26 (-0.89, 1.43)	-1.07 (-2.03, -0.11)	0.08 (-0.91, 1.09)	-0.51 (-1.37, 0.36)	-0.45 (-1.21, 0.31)
TV_{0-3} (°C)	-0.07 (-0.67, 0.54)	0.76 (-0.37, 1.91)	-0.89 (-1.83, 0.06)	0.47 (-0.51, 1.46)	-0.14 (-0.98, 0.72)	0.02 (-0.72, 0.77)
TV_{0-4} (°C)	0.32 (-0.29, 0.92)	0.96 (-0.17, 2.09)	-0.57 (-1.51, 0.37)	1.06 (0.08, 2.05)	0.34 (-0.50, 1.19)	0.31 (-0.43, 1.05)
TV_{0-5} (°C)	0.45 (-0.16, 1.06)	0.97 (-0.17, 2.11)	-0.56 (-1.50, 0.40)	1.01 (0.02, 2.01)	0.64 (-0.21, 1.50)	0.59 (-0.15, 1.33)
TV_{0-6} (°C)	0.58 (-0.04, 1.20)	1.06 (-0.09, 2.23)	-0.36 (-1.32, 0.61)	0.95 (-0.05, 1.97)	0.81 (-0.05, 1.68)	0.77 (0.02, 1.52)
TV_{0-7} (°C)	0.60 (-0.03, 1.23)	0.85 (-0.33, 2.04)	-0.22 (-1.21, 0.78)	0.89 (-0.13, 1.93)	0.87 (-0.01, 1.77)	0.80 (0.04, 1.57)
**Musculoskeletal system and connective tissue diseases**						
TV_{0-1} (°C)	-0.71 (-1.34, -0.07)	-0.43 (-1.94, 1.10)	-0.69 (-1.66, 0.30)	-1.10 (-2.07, -0.12)	-0.68 (-1.43, 0.07)	-0.38 (-1.01, 0.24)
TV_{0-2} (°C)	-0.63 (-1.24, -0.03)	-0.70 (-2.13, 0.74)	-0.44 (-1.36, 0.50)	-1.00 (-1.92, -0.06)	-0.61 (-1.32, 0.10)	-0.35 (-0.94, 0.24)
TV_{0-3} (°C)	-0.15 (-0.74, 0.45)	-0.38 (-1.78, 1.03)	0.21 (-0.71, 1.13)	-0.32 (-1.24, 0.60)	-0.27 (-0.97, 0.43)	0.02 (-0.55, 0.61)
TV_{0-4} (°C)	0.29 (-0.30, 0.88)	0.30 (-1.09, 1.72)	0.59 (-0.32, 1.50)	0.27 (-0.64, 1.20)	0.08 (-0.61, 0.78)	0.43 (-0.14, 1.00)
TV_{0-5} (°C)	0.29 (-0.30, 0.89)	0.42 (-0.99, 1.86)	0.27 (-0.65, 1.19)	0.40 (-0.53, 1.34)	0.00 (-0.69, 0.71)	0.66 (0.08, 1.23)
TV_{0-6} (°C)	0.37 (-0.23, 0.98)	0.64 (-0.80, 2.10)	0.12 (-0.81, 1.06)	0.51 (-0.43, 1.46)	0.08 (-0.63, 0.80)	0.82 (0.24, 1.41)
TV_{0-7} (°C)	0.33 (-0.29, 0.95)	0.72 (-0.75, 2.22)	-0.07 (-1.02, 0.89)	0.42 (-0.54, 1.40)	0.09 (-0.64, 0.82)	0.84 (0.24, 1.44)
**Symptoms, signs, and Ill-defined conditions**						
TV_{0-1} (°C)	-0.30 (-0.68, 0.09)	-0.23 (-0.90, 0.44)	-0.52 (-1.04, 0.00)	-0.32 (-0.89, 0.25)	-0.33 (-0.79, 0.14)	-0.08 (-0.45, 0.28)
TV_{0-2} (°C)	-0.47 (-0.83, -0.10)	-0.57 (-1.20, 0.06)	-0.73 (-1.23, -0.24)	-0.35 (-0.89, 0.19)	-0.36 (-0.80, 0.09)	-0.40 (-0.75, -0.06)
TV_{0-3} (°C)	-0.17 (-0.53, 0.19)	-0.35 (-0.97, 0.27)	-0.51 (-1.00, -0.03)	0.16 (-0.37, 0.70)	-0.07 (-0.50, 0.37)	-0.16 (-0.50, 0.18)
TV_{0-4} (°C)	0.14 (-0.22, 0.50)	-0.14 (-0.75, 0.48)	-0.30 (-0.78, 0.19)	0.48 (-0.05, 1.02)	0.24 (-0.19, 0.67)	0.27 (-0.07, 0.61)
TV_{0-5} (°C)	0.23 (-0.13, 0.59)	-0.13 (-0.75, 0.49)	-0.43 (-0.91, 0.06)	0.63 (0.09, 1.17)	0.32 (-0.11, 0.76)	0.54 (0.20, 0.88)
TV_{0-6} (°C)	0.29 (-0.07, 0.66)	-0.02 (-0.65, 0.61)	-0.46 (-0.96, 0.03)	0.75 (0.20, 1.30)	0.37 (-0.07, 0.82)	0.63 (0.28, 0.97)
TV_{0-7} (°C)	0.32 (-0.06, 0.69)	0.06 (-0.58, 0.71)	-0.50 (-1.00, 0.01)	0.74 (0.18, 1.30)	0.40 (-0.06, 0.85)	0.69 (0.34, 1.04)
**Injury and poisoning**						
TV_{0-1} (°C)	0.25 (-0.08, 0.57)	-0.06 (-0.77, 0.66)	0.53 (0.04, 1.02)	0.09 (-0.50, 0.69)	0.25 (-0.33, 0.84)	0.33 (-0.20, 0.86)
TV_{0-2} (°C)	0.06 (-0.25, 0.37)	-0.73 (-1.40, -0.05)	-0.06 (-0.53, 0.40)	-0.02 (-0.59, 0.55)	0.38 (-0.17, 0.93)	0.58 (0.08, 1.09)
TV_{0-3} (°C)	0.18 (-0.12, 0.49)	-0.91 (-1.57, -0.24)	-0.22 (-0.67, 0.24)	0.29 (-0.27, 0.85)	0.73 (0.19, 1.27)	0.87 (0.38, 1.36)
TV_{0-4} (°C)	0.29 (-0.02, 0.59)	-0.75 (-1.41, -0.09)	-0.12 (-0.57, 0.33)	0.58 (0.03, 1.14)	0.88 (0.34, 1.41)	0.78 (0.30, 1.26)
TV_{0-5} (°C)	0.26 (-0.04, 0.57)	-0.83 (-1.49, -0.16)	-0.18 (-0.64, 0.27)	0.62 (0.06, 1.18)	0.84 (0.30, 1.38)	0.80 (0.32, 1.29)
TV_{0-6} (°C)	0.22 (-0.09, 0.52)	-0.95 (-1.63, -0.27)	-0.16 (-0.62, 0.31)	0.64 (0.08, 1.22)	0.68 (0.13, 1.23)	0.75 (0.26, 1.25)
TV_{0-7} (°C)	0.26 (-0.05, 0.58)	-0.88 (-1.57, -0.19)	-0.03 (-0.50, 0.45)	0.73 (0.15, 1.32)	0.69 (0.13, 1.25)	0.68 (0.18, 1.18)

TV_{0-1}, temperature variability at 0–1 day; TV_{0-2}, temperature variability at 0–2 days; TV_{0-3}, temperature variability at 0–3 days; TV_{0-4}, temperature variability at 0–4 days; TV_{0-5}, temperature variability at 0–5 days; TV_{0-6}, temperature variability at 0–6 days; TV_{0-7}, temperature variability at 0–7 days.

Table S4. Season-specific percentage change in hospitalizations by causes associated with per interquartile increase in temperature variability (°C) in exposure days from 0–1 to 0–7 days in Hong Kong.

TV exposure	Percentage change in hospitalization (%) (95% CI)			
	Total	Cold seasons	Moderate seasons	Hot seasons
**All-cause hospitalization**				
TV_{0-1} (°C)	-0.41 (-0.92, 0.09)	-0.18 (-0.84, 0.49)	-0.31 (-1.56, 0.96)	-0.92 (-2.35, 0.53)
TV_{0-2} (°C)	-0.39 (-0.87, 0.09)	-0.25 (-0.85, 0.35)	-0.32 (-1.43, 0.81)	-0.78 (-2.06, 0.53)
TV_{0-3} (°C)	-0.01 (-0.48, 0.47)	0.01 (-0.56, 0.58)	0.09 (-0.96, 1.15)	-0.27 (-1.49, 0.97)
TV_{0-4} (°C)	0.36 (-0.11, 0.83)	0.31 (-0.24, 0.86)	0.45 (-0.55, 1.47)	0.21 (-0.97, 1.40)
TV_{0-5} (°C)	0.45 (-0.02, 0.93)	0.41 (-0.13, 0.96)	0.52 (-0.46, 1.51)	0.36 (-0.80, 1.53)
TV_{0-6} (°C)	0.50 (0.02, 0.98)	0.49 (-0.05, 1.03)	0.54 (-0.42, 1.51)	0.42 (-0.73, 1.57)
TV_{0-7} (°C)	0.51 (0.02, 1.00)	0.52 (-0.03, 1.06)	0.53 (-0.42, 1.50)	0.39 (-0.75, 1.55)
**Infectious and parasitic diseases**				
TV_{0-1} (°C)	-0.30 (-0.69, 0.09)	-0.22 (-0.73, 0.28)	-0.40 (-1.36, 0.57)	-0.19 (-1.28, 0.92)
TV_{0-2} (°C)	-0.49 (-0.86, -0.13)	-0.43 (-0.88, 0.03)	-0.58 (-1.44, 0.28)	-0.40 (-1.38, 0.60)
TV_{0-3} (°C)	-0.37 (-0.73, -0.01)	-0.37 (-0.8, 0.06)	-0.44 (-1.24, 0.37)	-0.21 (-1.15, 0.73)
TV_{0-4} (°C)	-0.05 (-0.41, 0.31)	-0.06 (-0.47, 0.36)	-0.12 (-0.89, 0.65)	0.14 (-0.76, 1.05)
TV_{0-5} (°C)	0.03 (-0.33, 0.39)	0.06 (-0.35, 0.47)	-0.06 (-0.81, 0.69)	0.24 (-0.64, 1.13)
TV_{0-6} (°C)	0.05 (-0.31, 0.42)	0.10 (-0.31, 0.51)	-0.05 (-0.79, 0.69)	0.24 (-0.63, 1.12)
TV_{0-7} (°C)	0.17 (-0.21, 0.54)	0.22 (-0.19, 0.64)	0.06 (-0.67, 0.80)	0.32 (-0.55, 1.20)
**Neoplasms**				
TV_{0-1} (°C)	-1.48 (-2.63, -0.31)	-0.89 (-2.4, 0.65)	-1.31 (-4.16, 1.63)	-2.53 (-5.74, 0.79)
TV_{0-2} (°C)	-0.92 (-2.02, 0.20)	-0.59 (-1.97, 0.81)	-0.78 (-3.34, 1.84)	-1.68 (-4.59, 1.33)
TV_{0-3} (°C)	0.01 (-1.09, 1.11)	0.10 (-1.21, 1.44)	0.16 (-2.26, 2.64)	-0.52 (-3.32, 2.35)
TV_{0-4} (°C)	0.74 (-0.35, 1.84)	0.73 (-0.55, 2.02)	0.88 (-1.45, 3.26)	0.41 (-2.30, 3.19)
TV_{0-5} (°C)	0.76 (-0.34, 1.88)	0.78 (-0.49, 2.05)	0.84 (-1.43, 3.16)	0.54 (-2.13, 3.27)
TV_{0-6} (°C)	0.88 (-0.24, 2.01)	0.95 (-0.32, 2.22)	0.89 (-1.35, 3.18)	0.67 (-1.97, 3.38)
TV_{0-7} (°C)	0.84 (-0.30, 2.00)	0.98 (-0.30, 2.27)	0.81 (-1.42, 3.10)	0.52 (-2.12, 3.22)
**Endocrine, nutritional, and metabolic diseases**				
TV_{0-1} (°C)	-0.23 (-0.70, 0.25)	0.03 (-0.58, 0.66)	-0.07 (-1.24, 1.12)	-0.90 (-2.24, 0.46)
TV_{0-2} (°C)	-0.32 (-0.77, 0.14)	-0.10 (-0.66, 0.46)	-0.21 (-1.25, 0.84)	-0.89 (-2.10, 0.33)
TV_{0-3} (°C)	-0.02 (-0.46, 0.43)	0.08 (-0.45, 0.61)	0.08 (-0.90, 1.07)	-0.44 (-1.59, 0.72)
TV_{0-4} (°C)	0.37 (-0.07, 0.81)	0.38 (-0.13, 0.89)	0.46 (-0.47, 1.41)	0.07 (-1.03, 1.19)
TV_{0-5} (°C)	0.53 (0.09, 0.97)	0.54 (0.04, 1.04)	0.60 (-0.31, 1.52)	0.28 (-0.80, 1.38)
TV_{0-6} (°C)	0.59 (0.14, 1.04)	0.61 (0.11, 1.11)	0.64 (-0.26, 1.54)	0.35 (-0.72, 1.43)
TV_{0-7} (°C)	0.58 (0.12, 1.03)	0.61 (0.11, 1.12)	0.62 (-0.27, 1.52)	0.32 (-0.75, 1.40)
**Blood and blood-forming organs diseases**				
TV_{0-1} (°C)	-0.47 (-1.10, 0.16)	-0.12 (-0.94, 0.71)	-0.44 (-2.00, 1.14)	-0.95 (-2.73, 0.85)
TV_{0-2} (°C)	-0.62 (-1.22, -0.02)	-0.31 (-1.05, 0.43)	-0.64 (-2.02, 0.76)	-1.04 (-2.64, 0.58)
TV_{0-3} (°C)	-0.28 (-0.86, 0.31)	-0.11 (-0.82, 0.59)	-0.28 (-1.58, 1.03)	-0.58 (-2.10, 0.96)
TV_{0-4} (°C)	0.22 (-0.36, 0.80)	0.30 (-0.38, 0.98)	0.22 (-1.02, 1.48)	0.02 (-1.44, 1.51)
TV_{0-5} (°C)	0.36 (-0.22, 0.95)	0.44 (-0.22, 1.12)	0.33 (-0.87, 1.56)	0.23 (-1.20, 1.68)
TV_{0-6} (°C)	0.38 (-0.22, 0.97)	0.47 (-0.19, 1.14)	0.32 (-0.87, 1.52)	0.27 (-1.14, 1.71)
TV_{0-7} (°C)	0.35 (-0.25, 0.96)	0.46 (-0.21, 1.14)	0.28 (-0.90, 1.47)	0.19 (-1.22, 1.62)
**Mental disorders**				
TV_{0-1} (°C)	-0.08 (-0.53, 0.37)	0.28 (-0.31, 0.88)	-0.12 (-1.25, 1.01)	-0.43 (-1.71, 0.87)
TV_{0-2} (°C)	-0.25 (-0.68, 0.17)	0.00 (-0.53, 0.54)	-0.29 (-1.29, 0.72)	-0.56 (-1.71, 0.6)
TV_{0-3} (°C)	-0.06 (-0.48, 0.36)	0.07 (-0.44, 0.58)	-0.08 (-1.01, 0.87)	-0.28 (-1.37, 0.82)
TV_{0-4} (°C)	0.24 (-0.18, 0.65)	0.31 (-0.18, 0.8)	0.23 (-0.66, 1.13)	0.09 (-0.96, 1.15)
TV_{0-5} (°C)	0.33 (-0.09, 0.75)	0.38 (-0.10, 0.86)	0.32 (-0.55, 1.20)	0.24 (-0.79, 1.28)
TV_{0-6} (°C)	0.36 (-0.06, 0.79)	0.40 (-0.08, 0.88)	0.35 (-0.51, 1.21)	0.31 (-0.70, 1.34)
TV_{0-7} (°C)	0.37 (-0.06, 0.81)	0.40 (-0.08, 0.89)	0.36 (-0.50, 1.22)	0.32 (-0.70, 1.34)
**Nervous system and sense organs diseases**				
TV_{0-1} (°C)	-0.61 (-1.43, 0.21)	0.00 (-1.07, 1.07)	-0.43 (-2.45, 1.63)	-1.71 (-3.99, 0.62)
TV_{0-2} (°C)	-0.37 (-1.14, 0.41)	0.07 (-0.9, 1.04)	-0.20 (-2.00, 1.63)	-1.36 (-3.42, 0.74)
TV_{0-3} (°C)	0.21 (-0.55, 0.98)	0.41 (-0.50, 1.34)	0.41 (-1.28, 2.13)	-0.60 (-2.56, 1.40)
TV_{0-4} (°C)	0.73 (-0.03, 1.49)	0.82 (-0.07, 1.71)	0.92 (-0.70, 2.57)	0.07 (-1.82, 2.00)
TV_{0-5} (°C)	0.74 (-0.02, 1.51)	0.82 (-0.06, 1.70)	0.88 (-0.70, 2.49)	0.17 (-1.69, 2.06)
TV_{0-6} (°C)	0.75 (-0.02, 1.53)	0.86 (-0.01, 1.74)	0.85 (-0.71, 2.43)	0.17 (-1.67, 2.04)
TV_{0-7} (°C)	0.70 (-0.10, 1.49)	0.84 (-0.04, 1.73)	0.76 (-0.79, 2.34)	0.03 (-1.80, 1.90)
**Circulatory system diseases**				
TV_{0-1} (°C)	-0.33 (-0.77, 0.11)	-0.13 (-0.70, 0.44)	-0.23 (-1.31, 0.87)	-0.83 (-2.07, 0.44)
TV_{0-2} (°C)	-0.31 (-0.73, 0.11)	-0.20 (-0.71, 0.32)	-0.21 (-1.18, 0.76)	-0.72 (-1.85, 0.41)
TV_{0-3} (°C)	0.02 (-0.39, 0.43)	0.02 (-0.47, 0.51)	0.13 (-0.78, 1.04)	-0.27 (-1.34, 0.81)
TV_{0-4} (°C)	0.37 (-0.03, 0.78)	0.32 (-0.15, 0.79)	0.48 (-0.38, 1.35)	0.19 (-0.83, 1.23)

TV_{0-5} (°C)	0.47 (0.06, 0.88)	0.41 (-0.05, 0.88)	0.56 (-0.29, 1.40)	0.34 (-0.66, 1.36)
TV_{0-6} (°C)	0.53 (0.12, 0.95)	0.49 (0.03, 0.96)	0.61 (-0.22, 1.44)	0.44 (-0.56, 1.44)
TV_{0-7} (°C)	0.56 (0.14, 0.98)	0.53 (0.06, 1.00)	0.62 (-0.20, 1.45)	0.44 (-0.55, 1.45)
**Respiratory system diseases**				
TV_{0-1} (°C)	0.09 (-0.24, 0.41)	0.20 (-0.21, 0.62)	0.02 (-0.77, 0.82)	0.06 (-0.86, 0.99)
TV_{0-2} (°C)	-0.07 (-0.38, 0.24)	-0.02 (-0.40, 0.35)	-0.13 (-0.83, 0.58)	-0.01 (-0.84, 0.83)
TV_{0-3} (°C)	0.14 (-0.16, 0.44)	0.16 (-0.20, 0.51)	0.11 (-0.56, 0.77)	0.20 (-0.59, 1.00)
TV_{0-4} (°C)	0.29 (0.00, 0.59)	0.29 (-0.05, 0.64)	0.27 (-0.36, 0.90)	0.39 (-0.37, 1.15)
TV_{0-5} (°C)	0.38 (0.08, 0.68)	0.39 (0.05, 0.73)	0.34 (-0.27, 0.96)	0.47 (-0.27, 1.22)
TV_{0-6} (°C)	0.45 (0.14, 0.75)	0.47 (0.13, 0.81)	0.40 (-0.21, 1.01)	0.53 (-0.21, 1.27)
TV_{0-7} (°C)	0.53 (0.22, 0.84)	0.57 (0.23, 0.91)	0.48 (-0.13, 1.09)	0.59 (-0.15, 1.33)
**Digestive system diseases**				
TV_{0-1} (°C)	-0.50 (-1.06, 0.05)	-0.52 (-1.23, 0.21)	-0.28 (-1.65, 1.10)	-0.95 (-2.50, 0.63)
TV_{0-2} (°C)	-0.40 (-0.92, 0.13)	-0.48 (-1.13, 0.17)	-0.20 (-1.42, 1.03)	-0.71 (-2.11, 0.71)
TV_{0-3} (°C)	-0.01 (-0.53, 0.5)	-0.19 (-0.81, 0.43)	0.20 (-0.94, 1.36)	-0.21 (-1.54, 1.14)
TV_{0-4} (°C)	0.31 (-0.21, 0.82)	0.09 (-0.51, 0.69)	0.52 (-0.57, 1.63)	0.21 (-1.07, 1.51)
TV_{0-5} (°C)	0.34 (-0.17, 0.86)	0.13 (-0.46, 0.72)	0.54 (-0.53, 1.62)	0.32 (-0.95, 1.59)
TV_{0-6} (°C)	0.41 (-0.11, 0.94)	0.24 (-0.35, 0.84)	0.58 (-0.47, 1.64)	0.39 (-0.86, 1.65)
TV_{0-7} (°C)	0.40 (-0.13, 0.94)	0.26 (-0.34, 0.86)	0.56 (-0.49, 1.62)	0.33 (-0.92, 1.59)
**Genitourinary system diseases**				
TV_{0-1} (°C)	-0.59 (-1.38, 0.20)	-0.42 (-1.45, 0.62)	-0.44 (-2.39, 1.55)	-1.12 (-3.33, 1.14)
TV_{0-2} (°C)	-0.61 (-1.36, 0.14)	-0.54 (-1.47, 0.39)	-0.48 (-2.22, 1.28)	-0.99 (-2.99, 1.04)
TV_{0-3} (°C)	-0.16 (-0.89, 0.58)	-0.29 (-1.17, 0.60)	0.01 (-1.62, 1.67)	-0.35 (-2.25, 1.59)
TV_{0-4} (°C)	0.34 (-0.39, 1.07)	0.15 (-0.71, 1.01)	0.52 (-1.04, 2.11)	0.27 (-1.56, 2.13)
TV_{0-5} (°C)	0.56 (-0.18, 1.30)	0.40 (-0.44, 1.25)	0.70 (-0.82, 2.25)	0.56 (-1.25, 2.39)
TV_{0-6} (°C)	0.55 (-0.19, 1.31)	0.43 (-0.41, 1.28)	0.66 (-0.85, 2.18)	0.59 (-1.19, 2.41)
TV_{0-7} (°C)	0.57 (-0.19, 1.34)	0.47 (-0.38, 1.33)	0.66 (-0.84, 2.18)	0.60 (-1.18, 2.41)
**Skin and subcutaneous tissue diseases**				
TV_{0-1} (°C)	-0.31 (-0.96, 0.34)	0.19 (-0.67, 1.05)	-0.40 (-2.02, 1.25)	-0.72 (-2.55, 1.15)
TV_{0-2} (°C)	-0.44 (-1.06, 0.18)	-0.11 (-0.89, 0.67)	-0.53 (-1.97, 0.93)	-0.72 (-2.37, 0.96)
TV_{0-3} (°C)	-0.07 (-0.67, 0.54)	0.07 (-0.67, 0.81)	-0.11 (-1.47, 1.26)	-0.19 (-1.76, 1.41)
TV_{0-4} (°C)	0.32 (-0.29, 0.92)	0.38 (-0.33, 1.10)	0.28 (-1.02, 1.59)	0.28 (-1.24, 1.81)
TV_{0-5} (°C)	0.45 (-0.16, 1.06)	0.54 (-0.16, 1.25)	0.36 (-0.90, 1.64)	0.47 (-1.02, 1.97)
TV_{0-6} (°C)	0.58 (-0.04, 1.20)	0.70 (0.00, 1.40)	0.45 (-0.80, 1.71)	0.63 (-0.83, 2.13)
TV_{0-7} (°C)	0.60 (-0.03, 1.23)	0.71 (0.01, 1.42)	0.46 (-0.77, 1.72)	0.67 (-0.79, 2.16)
**Musculoskeletal system and connective tissue diseases**				
TV_{0-1} (°C)	-0.71 (-1.34, -0.07)	-0.27 (-1.09, 0.57)	-0.53 (-2.09, 1.06)	-1.63 (-3.41, 0.17)
TV_{0-2} (°C)	-0.63 (-1.24, -0.03)	-0.31 (-1.06, 0.44)	-0.49 (-1.88, 0.92)	-1.43 (-3.03, 0.20)
TV_{0-3} (°C)	-0.15 (-0.74, 0.45)	0.02 (-0.69, 0.74)	-0.01 (-1.32, 1.32)	-0.79 (-2.31, 0.76)
TV_{0-4} (°C)	0.29 (-0.30, 0.88)	0.36 (-0.33, 1.05)	0.43 (-0.83, 1.70)	-0.21 (-1.68, 1.28)
TV_{0-5} (°C)	0.29 (-0.30, 0.89)	0.35 (-0.32, 1.04)	0.40 (-0.83, 1.64)	-0.16 (-1.60, 1.30)
TV_{0-6} (°C)	0.37 (-0.23, 0.98)	0.45 (-0.23, 1.13)	0.44 (-0.77, 1.66)	-0.06 (-1.48, 1.39)
TV_{0-7} (°C)	0.33 (-0.29, 0.95)	0.42 (-0.26, 1.11)	0.37 (-0.83, 1.59)	-0.13 (-1.56, 1.31)
**Symptoms, signs, and Ill-defined conditions**				
TV_{0-1} (°C)	-0.30 (-0.68, 0.09)	-0.23 (-0.73, 0.28)	-0.07 (-1.03, 0.91)	-0.85 (-1.94, 0.25)
TV_{0-2} (°C)	-0.47 (-0.83, -0.10)	-0.49 (-0.95, -0.03)	-0.26 (-1.12, 0.6)	-0.87 (-1.85, 0.12)
TV_{0-3} (°C)	-0.17 (-0.53, 0.19)	-0.27 (-0.70, 0.17)	0.03 (-0.77, 0.84)	-0.49 (-1.42, 0.45)
TV_{0-4} (°C)	0.14 (-0.22, 0.50)	0.02 (-0.40, 0.44)	0.32 (-0.44, 1.10)	-0.09 (-0.99, 0.81)
TV_{0-5} (°C)	0.23 (-0.13, 0.59)	0.13 (-0.29, 0.54)	0.39 (-0.36, 1.14)	0.05 (-0.83, 0.94)
TV_{0-6} (°C)	0.29 (-0.07, 0.66)	0.21 (-0.21, 0.62)	0.43 (-0.31, 1.17)	0.13 (-0.74, 1.00)
TV_{0-7} (°C)	0.32 (-0.06, 0.69)	0.25 (-0.17, 0.67)	0.44 (-0.29, 1.18)	0.12 (-0.75, 1.00)
**Injury and poisoning**				
TV_{0-1} (°C)	0.25 (-0.08, 0.57)	0.42 (-0.01, 0.85)	0.29 (-0.53, 1.11)	-0.05 (-0.98, 0.88)
TV_{0-2} (°C)	0.06 (-0.25, 0.37)	0.20 (-0.19, 0.58)	0.06 (-0.66, 0.79)	-0.17 (-1.00, 0.67)
TV_{0-3} (°C)	0.18 (-0.12, 0.49)	0.25 (-0.11, 0.62)	0.19 (-0.48, 0.88)	0.03 (-0.76, 0.82)
TV_{0-4} (°C)	0.29 (-0.02, 0.59)	0.32 (-0.03, 0.68)	0.29 (-0.35, 0.94)	0.18 (-0.57, 0.95)
TV_{0-5} (°C)	0.26 (-0.04, 0.57)	0.31 (-0.03, 0.66)	0.25 (-0.38, 0.88)	0.16 (-0.58, 0.91)
TV_{0-6} (°C)	0.22 (-0.09, 0.52)	0.28 (-0.07, 0.63)	0.19 (-0.43, 0.81)	0.12 (-0.62, 0.86)
TV_{0-7} (°C)	0.26 (-0.05, 0.58)	0.34 (-0.01, 0.70)	0.22 (-0.40, 0.85)	0.14 (-0.59, 0.88)

Cold seasons, months from December to February in next year; Hot seasons, months from July to September; Moderate seasons, months from March to May, October to November.

TV_{0-1}, temperature variability at 0–1 day; TV_{0-2}, temperature variability at 0–2 days; TV_{0-3}, temperature variability at 0–3 days; TV_{0-4}, temperature variability at 0–4 days; TV_{0-5}, temperature variability at 0–5 days; TV_{0-6}, temperature variability at 0–6 days; TV_{0-7}, temperature variability at 0–7 days.

Figure S1. Monthly trends from January to December of average temperature variabilities (°C) in exposure days from 0–1 to 0–7 days during 1999 to 2019 in Hong Kong.

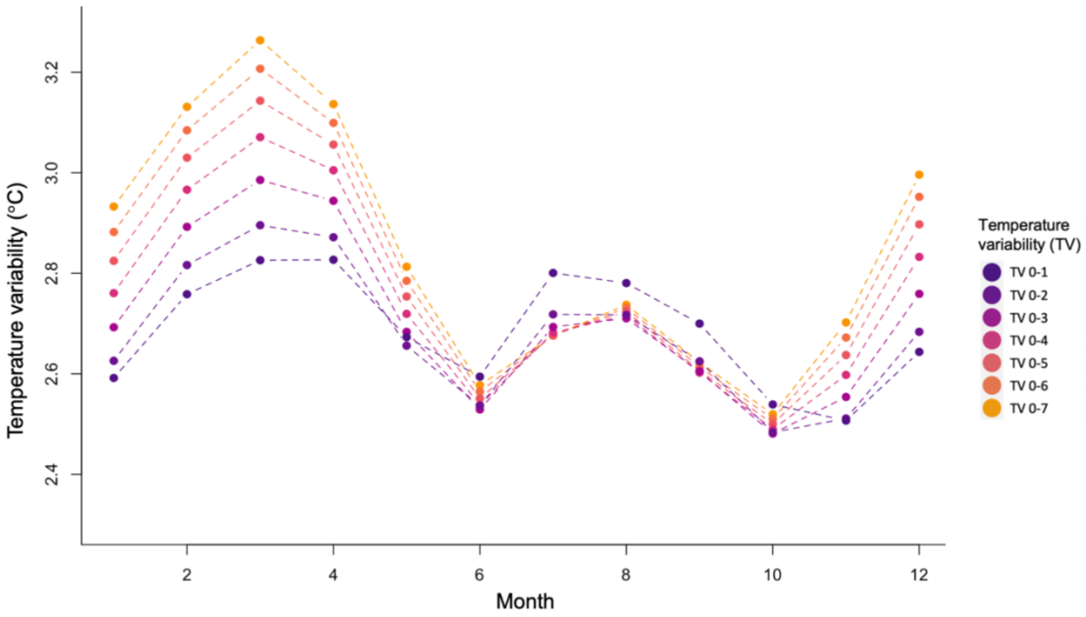

Supplement: Online Supplementary Document [file jogh-13-04122-s001.pdf]
